# Analysis on the Air-Gap Magnetic Field and Force of the Linear Synchronous Motor with Different Winding Distribution

**DOI:** 10.3390/mi16121396

**Published:** 2025-12-11

**Authors:** Jing Bai, Lei Zhang, Yu Xu

**Affiliations:** College of Electrical and Information Engineering, Beihua University, Jilin 132021, China

**Keywords:** PMLSM, pole-slot ratios, air gap flux density, analytical model, thrust

## Abstract

Based on the long-stator permanent magnet linear synchronous motor (PMLSM), motor structures with different pole–slot ratios are designed by changing the distribution of armature windings. A magnetic field analytical model of the motor is developed, the no-load magnetic field characteristics of the motor are calculated, and the results are compared and verified with those obtained by the finite element analysis (FEA). The influences of back-electromotive force (EMF) and armature reaction on the no-load magnetic field under different slots are studied. Through fast Fourier transform, the harmonic characteristics of the magnetic field in different structures are analyzed. Then, the cogging force and thrust characteristics generated by the motor in different structures are compared. The research results provide certain references for motor design.

## 1. Introduction

Linear motors directly convert electrical energy into linear motion through magnetic field coupling, avoiding the energy loss and precision degradation caused by the intermediate drive devices of rotary motors. This advantage is particularly significant in scenarios requiring long strokes and high speeds. Moreover, linear synchronous motors feature numerous advantages such as high thrust density, high power factor, and excellent dynamic performance, making them widely used in high-precision industrial assembly systems or high-speed propulsion systems [1,2]. With the advancement of permanent magnet materials and control technologies, their applications in fields like new energy vehicle drives and aerospace precision control are also continuously expanding.

Focused on the segmented-driven PMLSM, the thrust model and control equations of the motor are solved in [3], and the secondary actuator using the adaptive backstepping control method is shown to improve the tracking accuracy and robustness is confirmed. Based on the double-stator PMLSM, calculation and analysis models for the magnetic field and normal force of the motor are established, and the influence mechanism of end effects and armature reactions on normal force ripple are verified in [4]. In [5,6,7,8], various control strategies for PMLSM have been proposed and studied, improving the control accuracy of the motors. Meanwhile, the accuracy of the proposed strategies has been verified through tests on the bench. A variety of analytical methods have been proposed for the air-gap magnetic field and electromagnetic force of the PMLSM [9,10,11]. In order obtain the air-gap flux density and electromagnetic thrust, the hierarchical analytical model of multi-gap magnetic field of PMLSM is established, the air-gap flux density waveform distribution is analyzed, and the correctness of the analytical method is verified by finite element simulations [12]. By integrating the advantages of linear induction motors and permanent magnet linear motors, a novel long-primary double-sided linear flux-switching permanent magnet (DSLFSPM) motor is proposed in [13]. The topology, working principle, and electromagnetic performance of this motor are analyzed and compared with linear induction motors through finite element simulations. The results show that the proposed DSLFSPM motor has advantages such as higher efficiency, greater thrust, and smaller thrust ripple. In [14], the authors propose a comprehensive design and analysis method for the surface-mounted PMLSM, focusing on the impact of inductance modeling approaches on thrust force. It is verified that the look-up table (LUT) model can better reflect the nonlinear electromagnetic characteristics of the system and predict the motor’s thrust performance more accurately. This theory provides a new perspective for the research on the nonlinear electromagnetic characteristics of PMLSM. A novel frequency-modulated (FM) mover structure is investigated in [15] to address the issues arising from the large pole pitch of high-speed PMLSMs while avoiding damage to the thrust characteristics. The impacts of the FM secondary on the main magnetic field and permanent magnet eddy-current losses are analyzed using analytical methods and FEA, respectively. The electromagnetic thrust of the motor is also derived. In addition, an accurate analytical model of a double-sided air-core linear permanent magnet motor with segmented permanent magnet poles is presented in [16], where the thrust average and thrust ripple is precisely predicted by the proposed model. The back-electromotive force and flux density distribution of the motor are also determined by analytical and FEM methods. While, an improved magnetic equivalent circuit model of the iron-core linear permanent magnet synchronous motor for the flux density distribution and iron losses are predicted in [17]. Moreover, the magnetic saturation characteristics of the iron core are fully considered by utilizing nonlinear elements, and the effects of saturation and armature reaction on the flux density distribution are shown in detail.

At present, permanent magnet motors with fractional-slot concentrated windings have the advantages of small size and high efficiency, and have been widely used in fields such as aerospace and new energy vehicles [18,19]. However, in some linear motion fields, the stator structure of the motor is fixed, and the width of the stator core and the slot pitch cannot be changed. In this case, different pole–slot matching ratios can be formed by designing the energization sequence of the armature windings. There are few studies on the electromagnetic performance of this type of motor, and this paper focuses on this part of the content.

In this paper, first, the magnetic field analysis models for the permanent magnets and armature windings of the linear synchronous motor are established, respectively, and the distribution characteristics of the two types of magnetic fields are calculated using the analytical method. Then, the finite element models of the motor with a 9-slot/10-pole structure as well as a 12-slot/10-pole structure are built. The permanent magnet magnetic field is compared with the finite element results, which verified the effectiveness of the analytical method. After that, the influence of the armature magnetic field on the no-load magnetic field of the motor under different pole–slot configurations is analyzed. Additionally, harmonic analysis is conducted on the magnetic fields of the two types of motors, and their thrust characteristics are compared.

## 2. Structure and Operation Principle

### Modeling of the PMLSM

**Figure 1:**
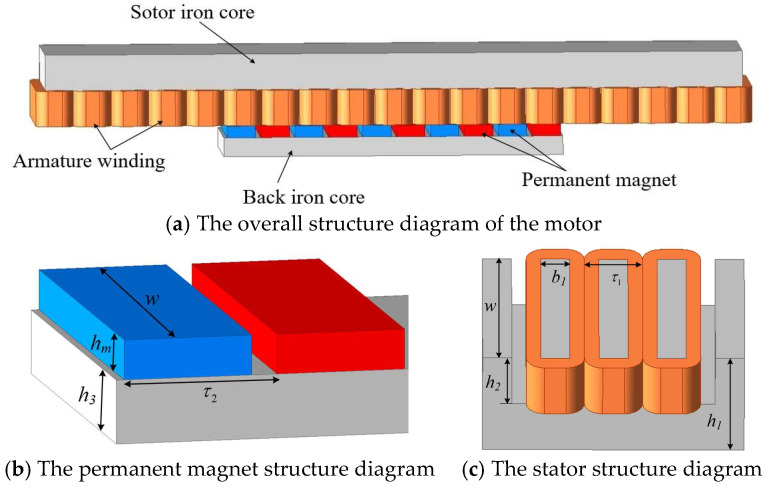
Structure diagram of the PMLSM.

The PMLSM adopts fractional-slot concentrated windings, which can reduce losses and minimize torque ripple. When different pole–slot combinations are used, the motor will exhibit different characteristics in terms of magnetic field and thrust. Therefore, in this paper, under the condition of the same number of poles, motors with 9-slot/10-pole as well as 12-slot/10-pole are established by only changing the number of stator slots. The 3D model of the motor is shown in Figure 1. The specific parameters of the motor are shown in Table 1.

**Figure 2:**
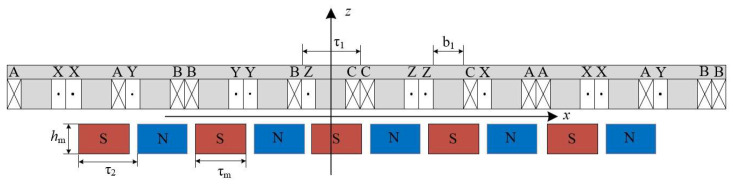
Analysis model in *x-z* plane.

As can be seen from Figure 1, the structure of a long stator and a short mover is adopted. Figure 2 shows the motor analysis model in the *x-z* plane. For the sake of simplifying calculations and analysis, the following assumptions are generally made:

(1) The stator and mover of the motor are infinitely long, and the models of each region extend infinitely along the *x*-axis;
(2) The transverse end effect is not considered, and the variation in the magnetic field along the *z*-axis is ignored;
(3) The magnetic permeability of the stator core and the mover yoke is infinite;
(4) Neglecting the leakage flux of windings and permanent magnets, the permanent magnets are uniformly magnetized.

This paper adopts the equivalent magnetization current method to calculate the distribution characteristics of the magnetic field of permanent magnets. According to the distribution function of permanent magnets, the Fourier series of the equivalent magnetization of the permanent magnet in the studied model can be expressed as

(1) $$M\left(x\right)=\sum_{n=1}^{2k-1}\frac{4B_{r}}{\mu_{0}n\pi }\sin\frac{n\pi }{2}\sin\frac{n\pi \tau_{m}}{2\tau_{2}}\sin\frac{n\pi }{\tau_{2}}x$$

where *k* = 1, 2, 3…, $\mu_{0}$ is the permeability of air, $B_{r}$ is the residual magnetization of the permanent magnet, τ is the polar distance, and $\tau_{m}$ is width of the permanent magnet.

The equivalent current density of the permanent magnet can be expressed as

(2) $$J_{m}\left(x\right)=\nabla \times M=\sum_{n=1}^{2k-1}\frac{4B_{r}}{\mu_{0}\tau }\sin\frac{n\pi }{2}\sin\frac{n\pi \tau_{m}}{2\tau }\cos\frac{n\pi }{\tau }x$$

**Figure 3:**
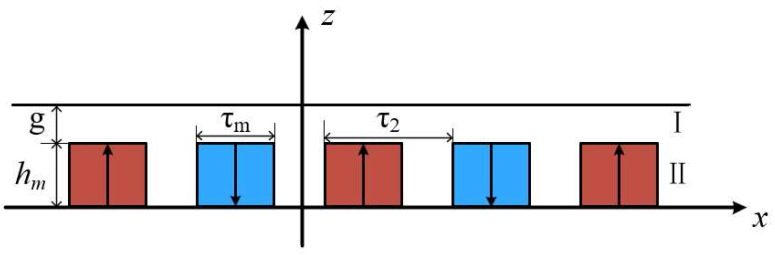
Subdomain analysis model of the PMLSM.

Figure 3 shows the subdomain analysis model of the PMLSM. Region I is the air region and Region II is the permanent magnet region. Based on the distribution of current density in Region I and Region II, the Poisson’s equations for the two regions can be written as

(3) $$\left\{\begin{array}{l} \frac{\partial^{2}A_{1}}{\partial x^{2}}+\frac{\partial^{2}A_{1}}{\partial z^{2}}=0\\ \frac{\partial^{2}A_{2}}{\partial x^{2}}+\frac{\partial^{2}A_{2}}{\partial z^{2}}=-\mu_{0}J_{m} \end{array}\right.$$

where $\mu_{0}$ is the permeability of air, $J_{m}$ is the equivalent current density of the permanent magnet, and *A*_1_ and *A*_2_ are the vector magnetic potentials of air-gap Region I and permanent magnet Region II, respectively.

The general solution of Equation (3) can be obtained by the method of separation of variables as follows:

(4) $$\left\{\begin{array}{l} A_{1}=\sum_{n=1}^{\infty }\left(K_{1}\cosh\left(\frac{n\pi }{\tau_{2}}z\right)+K_{2}\sinh\left(\frac{n\pi }{\tau_{2}}z\right)\right)\cos\frac{n\pi }{\tau }x\\ A_{2}=\sum_{n=1}^{\infty }\left(K_{3}\sinh\left(\frac{n\pi }{\tau_{2}}z\right)+K_{4}\cos\left(\frac{n\pi }{\tau_{2}}z\right)+M_{1}\right)\sin\frac{n\pi }{\tau }x \end{array}\right.$$

where $M_{1}=\frac{4B_{r}\tau_{2}}{n^{2}\pi^{2}}\sin\frac{n\pi }{2}\sin\frac{n\pi \tau_{m}}{2\tau_{2}}$, *K*_1_, *K*_2_, *K*_3_, and *K*_4_ are the coefficients to be solved and $\tau_{2}$ is the pole pitch of the permanent magnet.

According to the continuity boundary conditions of the magnetic field, the magnetic field intensity and magnetic flux density at different positions in the air-gap region and the permanent magnet region satisfy

(5) $$\left\{\begin{array}{l} \begin{array}{cc} y=0, & H_{x2}=0 \end{array}\\ y=h_{m}\;H_{x1}=H_{x2}\;B_{y1}=B_{y2}\\ \begin{array}{cc} y=g+h_{m}, & H_{x1}=0 \end{array} \end{array}\right.$$

where *g* is the air-gap size, $h_{m}$ is the permanent magnet height, $H_{x1}$ and $H_{x1}$ are the magnetic field intensities in different regions, and $H_{x1}$ and $H_{x1}$ are the magnetic flux densities in different regions.

Based on Equations (4) and (5), the expressions for the magnetic field in the air-gap region and the permanent magnet region can be derived as follows:

(6) $$\left\{\begin{array}{l} B_{x1}=\sum_{n=1}^{\infty }\frac{n\pi }{\tau }\left(C_{1}\cosh\left(\frac{n\pi }{\tau }z\right)+C_{2}\sinh\left(\frac{n\pi }{\tau }z\right)\right)\cos\frac{n\pi }{\tau }x\\ B_{y1}=-\sum_{n=1}^{\infty }\frac{n\pi }{\tau }\left(C_{1}\sinh\left(\frac{n\pi }{\tau }z\right)+C_{2}\cosh\left(\frac{n\pi }{\tau }z\right)\right)\sin\frac{n\pi }{\tau }x \end{array}\right.$$

(7) $$\left\{\begin{array}{l} B_{x2}=\sum_{n=1}^{\infty }\frac{n\pi }{\tau }\left(C_{3}\cosh\left(\frac{n\pi }{\tau }z\right)+C_{4}\sinh\left(\frac{n\pi }{\tau }z\right)\right)\cos\frac{n\pi }{\tau }x\\ B_{y2}=-\sum_{n=1}^{\infty }\frac{n\pi }{\tau }\left(C_{3}\sinh\left(\frac{n\pi }{\tau }z\right)+C_{4}\cosh\left(\frac{n\pi }{\tau }z\right)+D_{1}\right)\sin\frac{n\pi }{\tau }x \end{array}\right.$$

where

(8) $$\left\{\begin{array}{c} C_{1}=-D_{1}\sinh\left(\frac{n\pi h_{m}}{\tau }\right)\\ C_{2}=D_{1}\sinh\left(\frac{n\pi h_{m}}{\tau }\right)\coth\left(\frac{n\pi \left(g+h_{m}\right)}{\tau }\right)\\ C_{3}=0\\ C_{4}=-D_{1}\frac{\sinh\left(\frac{n\pi g}{\tau }\right)g}{\sinh\left(\frac{n\pi \left(g+h_{m}\right)}{\tau }\right)} \end{array}\right.$$

## 3. Model Verification and Result Analysis

**Figure 4:**
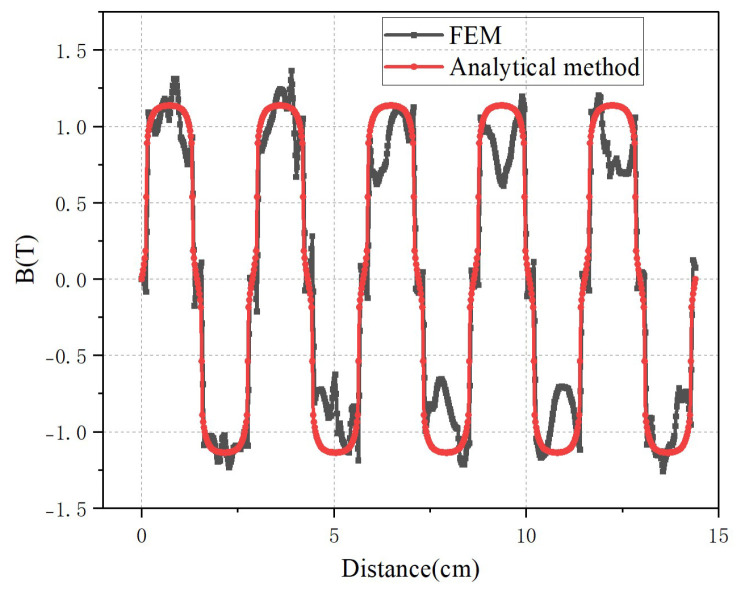
The air-gap magnetic flux density with 9-slot/10-pole structure for two methods.

Figure 4 shows the comparison of the motor’s air-gap flux density obtained by the analytical method and the FEA. As can be seen from the figure, the variation trends of the air-gap flux density obtained by the analytical method and that by the FEA are basically consistent. However, there is a certain difference in the amplitude of the flux density. This is because, in the analytical calculation, the permanent magnet is assumed to be uniformly magnetized, and the leakage flux of the stator and mover is not considered. In addition, the magnetic field shows a depression at the maximum or minimum flux density. This is due to the influence of the stator slotting: at the slots, the air-gap reluctance increases, leading to a reduction in the air-gap flux density.

The assumption that the permeability of the stator and mover yoke is infinite neglects the magnetic saturation effect and the magnetomotive force (MMF) drop that actually exists in the iron-core magnetic circuit. In reality, the permeability of the iron core is finite and exhibits nonlinear characteristics. This assumption causes the analytical model to overestimate the amplitude of the air-gap magnetic flux density, as it defaults to the notion that all MMF generated by the permanent magnets acts solely on the air-gap. In contrast, the FEA takes into account the nonlinear B-H curve of the iron-core material, enabling it to more accurately reflect the true MMF distribution. Consequently, the amplitude of the magnetic flux density obtained from FEA is slightly lower and more consistent with real-world conditions.

The assumption of infinite core permeability, while enabling a tractable analytical solution, inherently neglects magnetic saturation and the nonlinear *B-H* characteristic of the lamination material. Future refinements of this model could incorporate an effective permeability derived from the material’s *B* = *f*(*H*) curve or couple with a nonlinear magnetic equivalent circuit to better predict performance under higher loading or saturated conditions.

**Figure 5:**
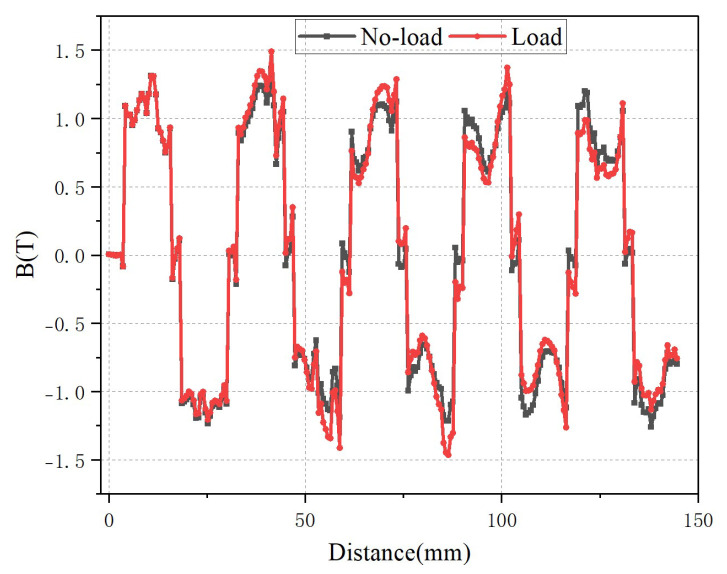
The air-gap magnetic flux density of 9-slot/10-pole.

Figure 5 presents a comparative analysis of the variation in air-gap flux density of the 9-slot/10-pole motor under no-load and load conditions. Due to the effect of the armature magnetic field, at the top part of the maximum flux density generated by the permanent magnet, a phenomenon occurs where the flux density decreases on one side and increases on the other. This is caused by the superposition of the alternating positive and negative armature magnetic field and the permanent magnet magnetic field. However, the increase in the amplitude of the no-load magnetic field generated by the permanent magnet is small; the maximum magnetic field increases from 1.36 T to approximately 1.5 T. In general, the armature magnetic field has little influence on the permanent magnet magnetic field, which is conducive to achieving precise control of the motor by adjusting the stator current.

**Figure 6:**
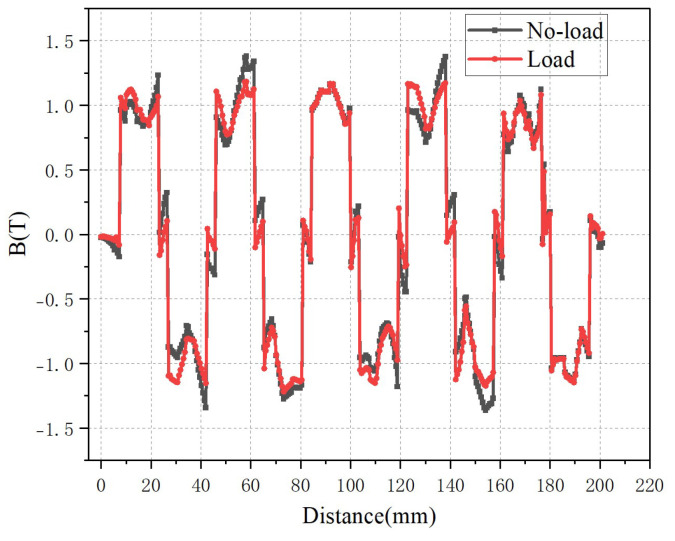
The air-gap magnetic flux density of 12-slot/10-pole.

Figure 6 shows the variation in air-gap flux density of the 12-slot/10-pole motor under no-load and load conditions. The armature magnetic field causes the flux density generated by the permanent magnet to decrease on the right side and increase on the left side, which is the opposite of the phenomenon in the 9-slot/10-pole motor. Although the number of poles of the two types of motors remains unchanged, when different numbers of slots are adopted, the energization sequence of the three-phase windings of the motors changes, resulting in different distribution characteristics of the armature magnetic field.

Due to the superposition of the alternating positive and negative armature magnetic field and the permanent magnet magnetic field, the amplitude of the no-load magnetic field generated by the permanent magnets decreases, and the maximum magnetic field drops from 1.36 T to approximately 1.2 T. Similarly to the case in Figure 5, overall, the armature magnetic field has little influence on the permanent magnet magnetic field.

From the comparison of the results in Figure 5 and Figure 6, it can be seen that with the same number of poles and without changing the stator pole pitch, the 9-slot motor has a larger amplitude of flux density. Meanwhile, the 9-slot motor exhibits a smaller armature reaction, which enables better realization of rotor flux orientation control in some fields requiring precise control. To more accurately analyze the characteristics of the air-gap magnetic fields of the two types of motors, the fast Fourier transform (FFT) is performed on the magnetic field by using one pair of pole data.

**Figure 7:**
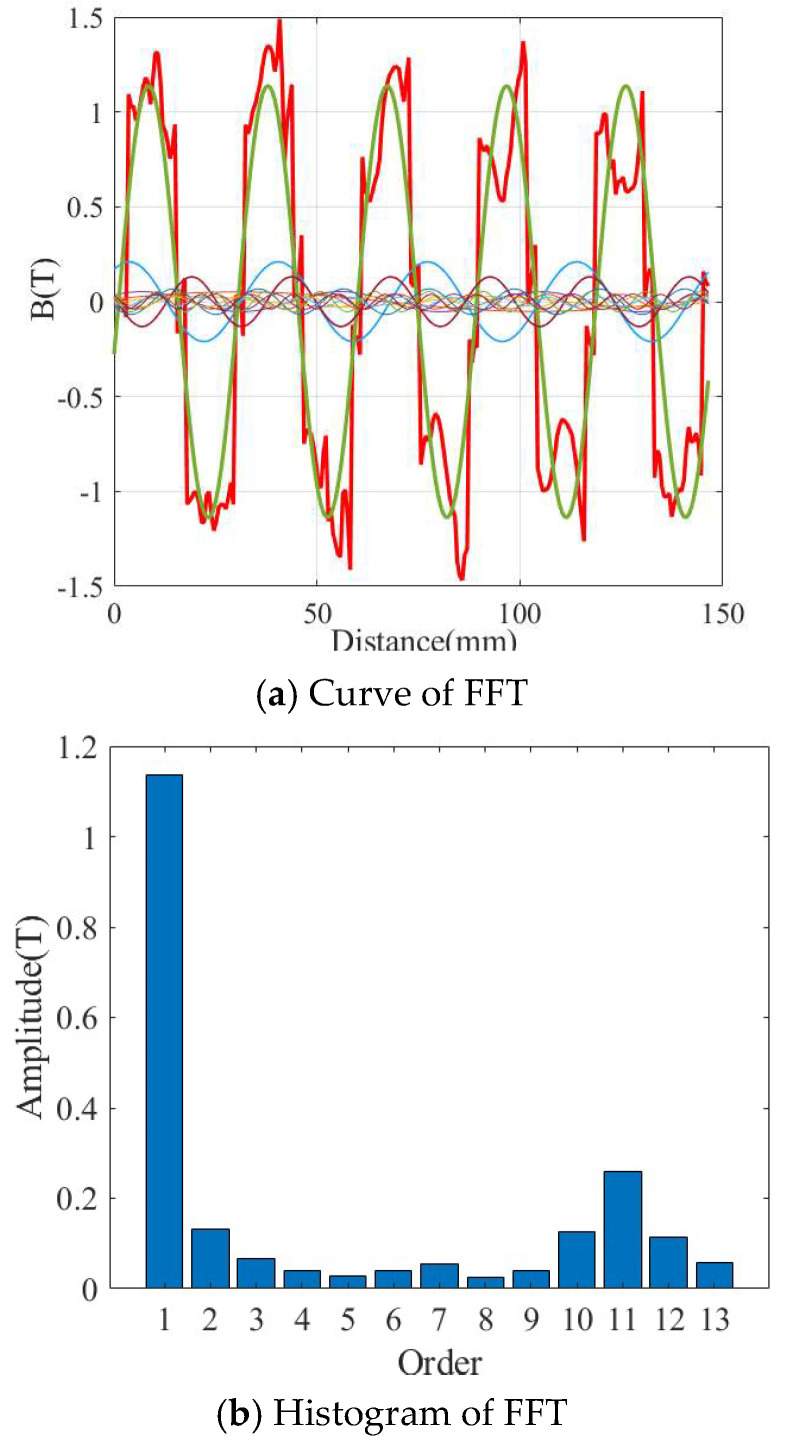
FFT of the air-gap flux density of 9-slot/10-pole.

Figure 7 shows the FFT diagram of the air-gap magnetic field of the 9-slot/10-pole motor. The 13 harmonics of the air-gap magnetic field are analyzed using MATLAB (2023a). It can be seen that, affected by the stator slotting, no flat-top wave appears at the top of the magnetic field, and the amplitude of the fundamental wave (the blue part) is approximately 1.3 T. As shown in Figure 7b, the amplitude of the 11th harmonic is relatively large, reaching 0.3 T, while the amplitude of the 3rd and 13th harmonic are about 0.06 T. The 3rd and the 13th harmonic account for 5.3% of the amplitude of the fundamental wave, and the proportion of the 10th and 12th harmonics reaches 9.7%. but the 11th harmonics account for 22% of the fundamental wave. The amplitudes of other harmonics are all less than 0.05 T, which can be ignored.

**Figure 8:**
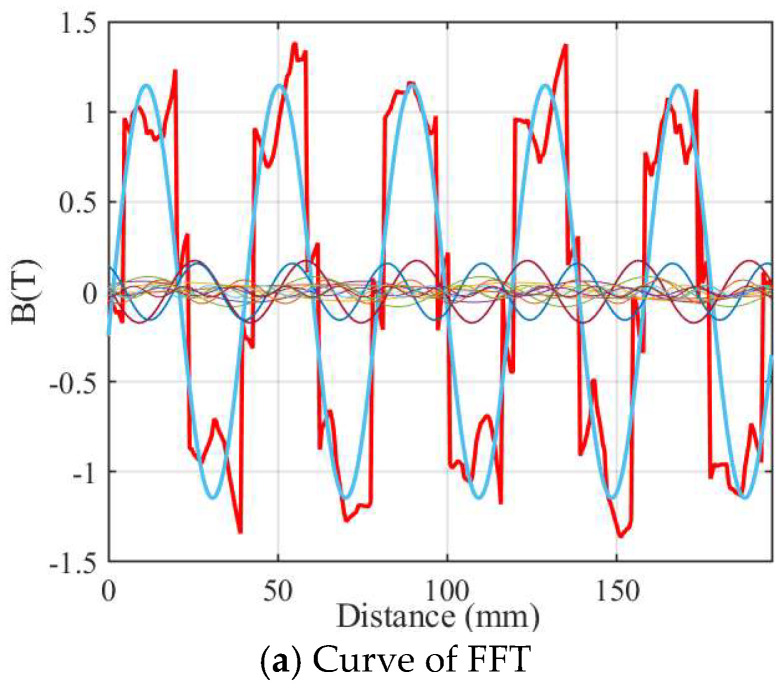

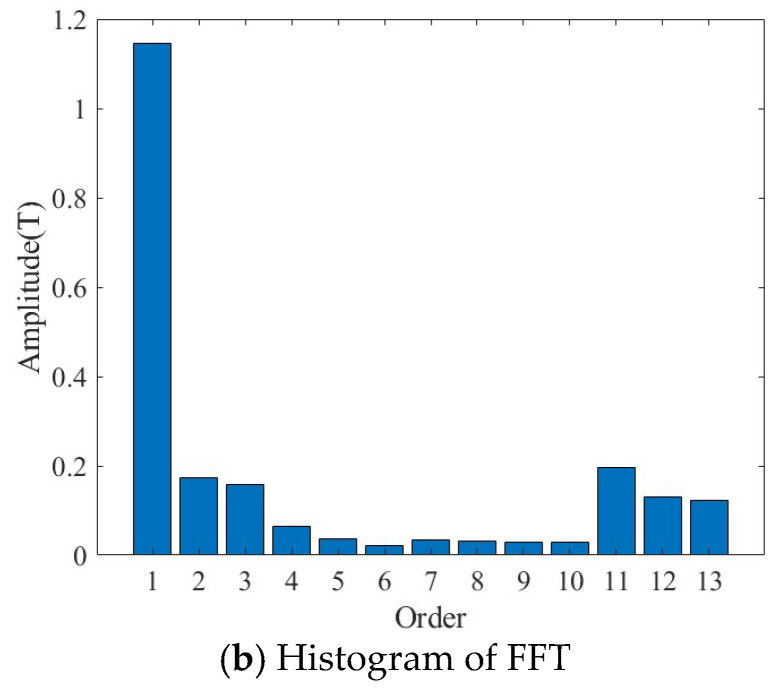
FFT of the air-gap flux density of 12-slot/10-pole.

Figure 8 shows the FFT diagram of the air-gap magnetic field of the 12-slot/10-pole motor. It can be clearly seen that the amplitude of the air-gap magnetic field of the 12-slot motor is significantly smaller than that of the 9-slot motor shown in Figure 7a. Similarly, affected by the stator slotting, a depression appears at the top of the air-gap magnetic field of the 12-slot motor. The amplitude of the fundamental wave (the blue part) is approximately 1.12 T, which is slightly smaller than that of the 9-slot motor. As shown in Figure 8b, the 11th harmonic reaches 0.19 T, which is 0.11 T smaller than that of the 9-slot motor. The amplitude of the 3rd harmonic is 0.15 T, and the amplitude of the 13th harmonic is about 0.12 T. The 5th–9th harmonics are basically very small which is the same as those of the 9-slot motor.

However, in the 12-slot motor, the amplitude of the 3rd harmonic increases significantly, reaching nearly 0.16 T. The 3rd harmonic accounts for 14% of the amplitude of the fundamental wave, the 11th and 13th harmonics account for 17% and 11% of the amplitude of the fundamental wave, respectively, and the 5th harmonic accounts for about 3.2% of the fundamental wave. It can be seen that there are far fewer harmonics in the 12-slot structure.

Although the aforementioned assumptions introduce quantifiable errors, it is important to emphasize that the impact of these errors is essentially negligible. The comparison results between the 9-slot/10-pole and 12-slot/10-pole structures remain valid and valuable for reference. The conclusion that the 9-slot structure generates a higher fundamental magnetic flux density is reliable. Under the same no-load conditions, due to the low level of iron-core saturation, the overestimation ratios of the flux density amplitude for the two structures are likely to be similar.

Therefore, this analytical model still holds significant value in the initial design phase and parameter analysis of the motor. Before conducting FEA, which requires greater computational resources, this model can be used to quickly and intuitively evaluate the influence of different slot-pole combinations on the trends of electromagnetic performance.

**Figure 9:**
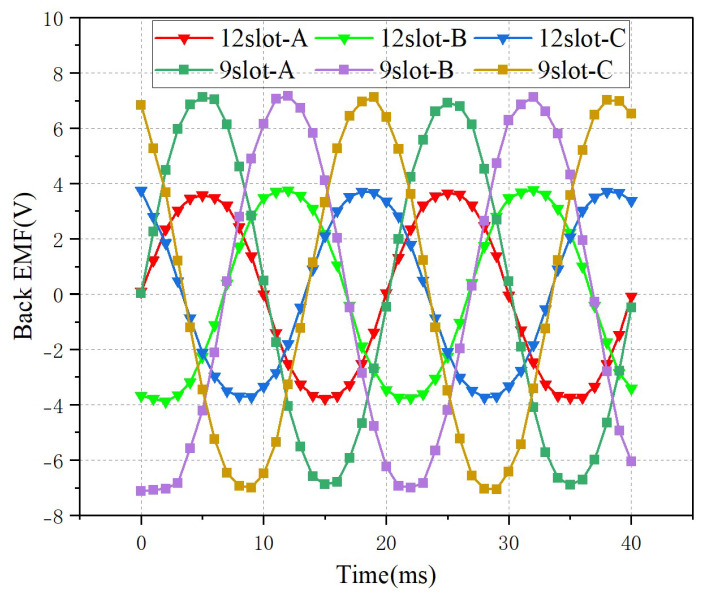
Back EMF of the motor with different slots.

Figure 9 is a comparison diagram of the back-electromotive force (EMF) of the two types of motors. As can be seen from the diagram, the EMF waveforms of both types of motors show a sinusoidal distribution, and the waveforms are good. Among them, the maximum value of the electromotive force of the 9-slot motor reaches about 4 V, and that of the 12-slot motor is about 7 V. The thickness of the two motors in the longitudinal reverse direction is the same. The EMF is proportional to the flux density and the speed of the mover. In the structure of the 12-slot motor, to keep the stator pole pitch of the motor unchanged, the pole pitch and mover speed of the motor are larger than those of the 9-slot motor. Therefore, the no-load EMF of the 12-slot motor increases.

**Figure 10:**
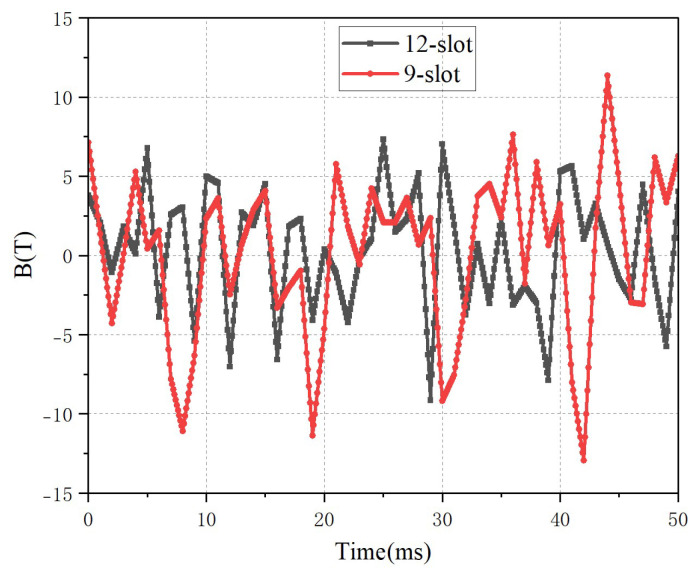
Cogging force of the motor with different slots.

For the two types of motors, the cogging force is calculated over two cycles of motor movement, and the results are shown in Figure 10. With the stator structure, the maximum cogging force of the 9-slot motor reaches approximately 12.5 N, while that of the 12-slot motor is about 9 N. According to the calculation properties and formula of cogging force, and based on the magnetic field calculation results obtained from Figure 5 and Figure 6, it can be known that the 9-slot motor has a larger amplitude of the no-load magnetic field, and thus generates a larger cogging force.

**Figure 11:**
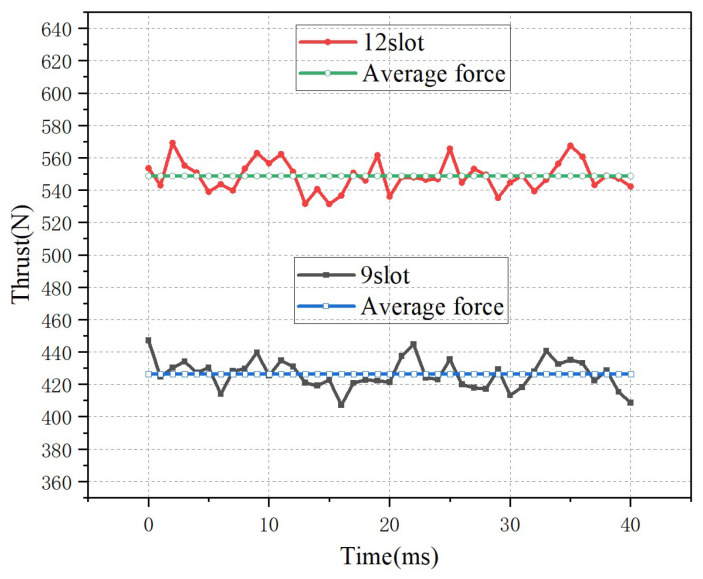
The thrust of the motor with different slots.

The thrust calculation results of the motors with different slot numbers are shown in Figure 11. The average thrust of the 12-slot motor is 549 N, the maximum thrust fluctuation reaches 569 N, and the thrust fluctuation is 3.6%. For the 9-slot motor, the average thrust is 425 N, the maximum thrust fluctuation reaches 445 N, and the thrust fluctuation is 4.7%. Overall, the thrust fluctuations of both types of motors are very small, both less than 5%. According to the previous calculation results of the cogging force, the 9-slot motor has a larger cogging force, so the thrust fluctuation of the motor is slightly increased.

**Figure 12:**
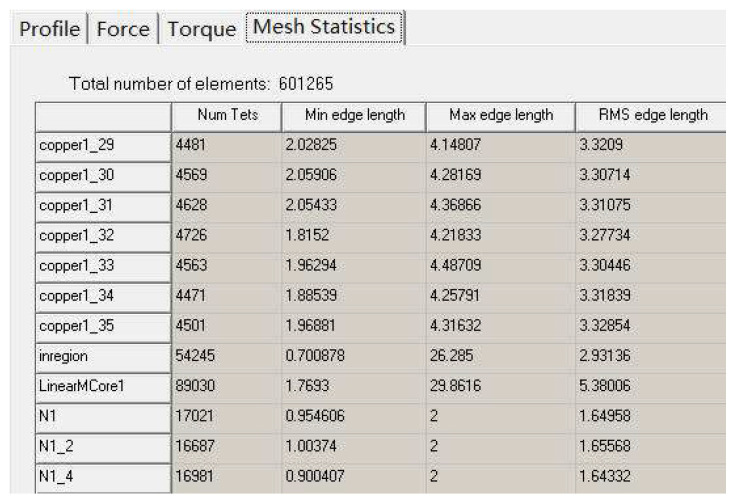
The elements for the motor.

The discretization mesh of the motor FEA simulation is presented and the information about the discretization mesh including number of nodes and number of elements is shown in Figure 12, where copper 1_29 and copper 1_30 are three-phase windings and N1, N1_2, and N1_4 are permanent magnet poles.

A detailed performance comparison is shown in Table 2 that clearly lists the advantages and disadvantages of the two motor structures across key performance indicators.

## 4. Conclusions

The analytical model for the air-gap magnetic field of the motor is established and the magnetic field distribution of the motor is calculated. The magnetic field characteristics of the motor with different pole–slot ratios are analyzed using the FEA and the analytical method. A detailed study is conducted on the harmonics of the air-gap magnetic field generated by the two types of motors. Finally, the cogging force and thrust of the motors are compared and analyzed. On the basis of not changing the stator size of the motor, different pole–slot combination structures are formed by changing the energization sequence of the armature windings. In this case, the 9-slot/10-pole motor has better magnetic field performance, but the cogging force and thrust fluctuation will also increase slightly. It is of certain significance for motor design in occasions where it is not easy to modify the motor stator size.

However, the proposed analytical model offers the advantage of fast computation and is well-suited as a basis for further design optimization of the motor. Future research can integrate this model with optimization strategies such as the Taguchi method [20,21] to efficiently determine the optimal winding distribution and pole–slot combination under given constraints, thereby comprehensively enhancing the overall performance of the motor.

## Figures and Tables

**Table 1:** The main parameters of the motor.

Symbol	Quantity	Value
*τ*	Pole pitch of 9-slot/12-slot	14.4/19.2 mm
*g*	Air-gap	0.5 mm
*I*_1_	Stator phase current	100 A
*N*_1_	Stator phase current turns	50
*h*_m_	Permanent magnet height	5 mm
*τ*_1_	Stator slot pitch	12mm
*τ*_2_	Pole length	16 mm
*b_1_*	Stator tooth width	8 mm
*w*	Permanent magnet width	50 mm
*h_1_*	Stator iron height	30 mm
*h_2_*	Iron tooth height	15 mm
*h_3_*	Permanent magnet backplate height	8 mm

**Table 2:** The comparison results of different motors.

Performance Metric	9-slot/10-pole	12-slot/10-pole	Analysis and Discussion
Air-gap Flux Density Fundamental (T)	1.3 T	1.12 T	The 9-slot structure has a higher flux density, which is beneficial for improving thrust density.
11th Harmonic (T)	0.3 T	0.19 T	The 12-slot structure is superior in suppressing specific harmonics.
Cogging Force (N)	12.5 N	9 N	The 12-slot structure has a smaller cogging force, which is conducive to stable operation.
Average Thrust (N)	425 N	549 N	The 12-slot structure provides greater average thrust.
Thrust Ripple Rate	4.7%	3.6%	The 12-slot structure achieves more stable thrust.
Back-EMF (V)	4 V	7 V	The 12-slot structure has a higher back-EMF.

## Data Availability

The original contributions presented in this study are included in the article. Further inquiries can be directed to the corresponding author.
